# *Klebsiella pneumonia* liver abscess causing bacteremia, pneumonia, and secondary brain abscess: A case report

**DOI:** 10.1097/MD.0000000000046803

**Published:** 2025-12-19

**Authors:** BoLin Wang, MingZhi Fan, FeiHu Zhang, Hao Zhao, PengCheng Li, Lei Zhou

**Affiliations:** a Affiliated Hospital of Shandong University of Traditional Chinese Medicine (First Clinical Medical College of Shandong University of Traditional Chinese Medicine), Jinan City, Shandong Province, China; b Shandong Provincial Police General Hospital, Jinan City, Shandong Province, China.

**Keywords:** bacteremia, brain abscess, case report, *Klebsiella pneumoniae*, liver abscess

## Abstract

**Rationale:**

Hypervirulent *Klebsiella pneumoniae* (hvKp) can cause an invasive syndrome characterized by multi-organ abscesses. While liver abscess and bacteremia are common, secondary brain abscess is a rare but life-threatening complication. The purpose of this case report is to illustrate the diagnostic and therapeutic challenges in managing such severe KPIS cases and to emphasize key management strategies that can lead to successful outcomes, thereby enhancing clinician awareness of this complex syndrome.

**Patient concerns:**

A 37-year-old male presented with fever and altered mental status, which subsequently progressed to septic shock and hypoxemia, requiring intensive care.

**Diagnoses:**

KPIS with liver abscess, bacteremia, pneumonia, and secondary brain abscess due to hvKp infection.

**Interventions:**

The patient received meropenem via extended infusion, fluid resuscitation, glycemic control, and supportive care.

**Outcomes:**

The patient’s condition improved significantly within 5 days of targeted therapy, manifested by the resolution of fever, successful extubation, and normalization of key laboratory parameters (including white blood cell count, C-reactive protein, and procalcitonin). He was discharged from the ICU after 15 days, exhibiting only mild residual right-sided weakness. Crucially, follow-up imaging at 3 months revealed complete resolution of the liver and brain abscesses, with no evidence of recurrence.

**Lessons:**

This case underscores the importance of early recognition, aggressive antimicrobial therapy, glycemic control, and the utility of next-generation sequencing in diagnosing hvKp infections. Strict glycemic management and susceptibility-guided therapy are critical to preventing complications and improving outcomes.

## 1. Introduction

*Klebsiella pneumoniae* invasive syndrome (KPIS) is a systemic multiorgan infection caused by hypervirulent strains of *Klebsiella pneumoniae*. It is typically characterized by primary liver abscess accompanied by bacteremia and metastatic infections such as brain abscess, purulent meningitis, endophthalmitis, and necrotizing fasciitis.^[1]^ KPIS predominantly affects immunocompromised individuals, including those with diabetes mellitus or malignancies, with a notably higher incidence among diabetic patients. This increased susceptibility is likely due to impaired immune function and enhanced vascular permeability, which facilitate bacterial invasion and dissemination.

Although KPIS has gained increasing recognition within the medical community, brain abscesses specifically caused by hypervirulent *K. pneumoniae* (hvKp) remain relatively uncommon. This report describes a case of KPIS due to hvKp infection with multi-organ involvement to raise clinical awareness and understanding of this severe condition.

## 2. Case description

### 2.1. Patient characteristics

A 37-year-old male was admitted to the emergency department on February 18, 2025, with a 2-day history of fever and altered mental status. On presentation, his body temperature was 39.0°C, pulse was 100 beats per minute, and he reported chills, rigors, and low back pain. The patient denied cough, sputum production, chest tightness, or dyspnea. Initially evaluated at a local clinic, he was suspected of having epidemic hemorrhagic fever and was subsequently transferred to our hospital. Upon admission, he developed shock and hypoxemia, necessitating mechanical ventilation and immediate transfer to the intensive care unit (ICU).

### 2.2. Clinical findings

At admission, vital signs were as follows: temperature 39.1°C, blood pressure 129/79 mm Hg, heart rate 100 bpm, respiratory rate 18 breaths per minute, and blood glucose 18.2 mmol/L. The patient was confused, restless, and exhibited persistent high-grade fever, profuse sweating, tachypnea, and apparent distress. Physical examination of the chest and abdomen revealed no significant abnormalities.

### 2.3. Diagnostic evaluation

Arterial blood gas analysis indicated metabolic and respiratory acidosis: pH 7.264, PCO₂ 43.1 mm Hg, PO₂ 119 mm Hg, Na⁺ 132 mmol/L, K⁺ 5.1 mmol/L, Ca²⁺ 1.04 mmol/L, Cl⁻ 110 mmol/L, glucose 18.2 mmol/L, bicarbonate 19.5 mmol/L, total hemoglobin 14.4 g/dL, lactate 1.8 mmol/L, base excess −7.3 mmol/L, and anion gap 2.6 mmol/L. Complete blood count showed: white blood cells (WBC) 9.86 × 10⁹/L, hemoglobin 142 g/L, platelets 7.00 × 10⁹/L, and neutrophils 89.70%.Biochemical testing revealed: blood urea nitrogen 9.2 mmol/L, creatinine 136 μmol/L, C-reactive protein 194.6 mg/L, albumin 30.4 g/L, and procalcitonin 49.75 ng/mL. Liver function tests were notable for: alanine aminotransferase 226 U/L, aspartate aminotransferase 169 U/L, total bilirubin 37.7 μmol/L, and direct bilirubin 10.8 μmol/L. Urinalysis showed ketones (+) and white blood cells 45.1/μL.

**Figure 1.**
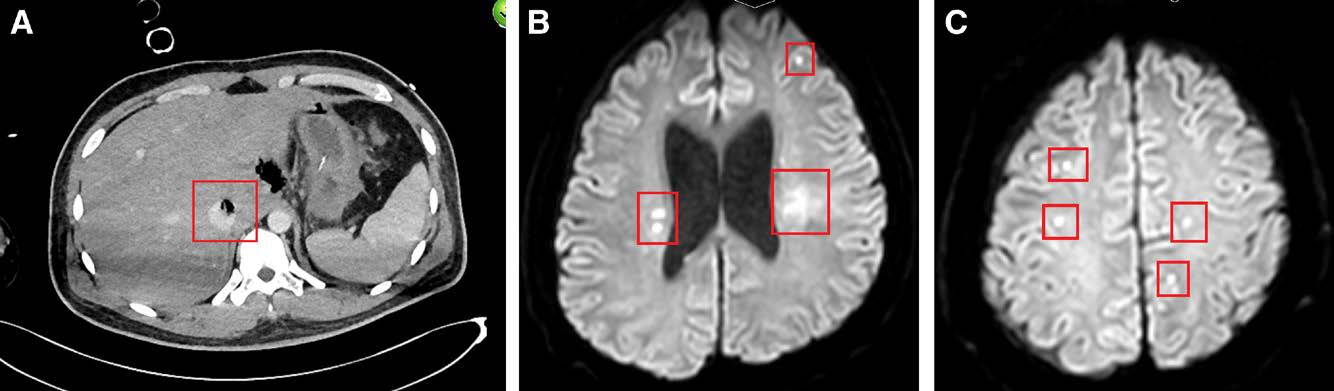
(A) Contrast-enhanced computed tomography (CT) of the chest, abdomen, and pelvis demonstrated patchy hypodense areas with gas formation in the caudate lobe of the liver and adjacent abdominal cavity, along with portal vein thrombosis. (B,C) Magnetic resonance imaging (MRI) of the brain revealed multiple abnormal signals in both cerebral hemispheres and bilateral ventricles.

Contrast-enhanced computed tomography (CT) of the chest, abdomen, and pelvis demonstrated patchy hypodense areas with gas formation in the caudate lobe of the liver and adjacent abdominal cavity, along with portal vein thrombosis (Fig. 1A). Magnetic resonance imaging (MRI) of the brain revealed multiple abnormal signals in both cerebral hemispheres and bilateral ventricles (Fig. 1B and 1C). Ophthalmologic examination showed no signs of endophthalmitis.

Microbiological cultures of sputum, urine, blood, cerebrospinal fluid, and liver abscess aspirate grew *Klebsiella pneumoniae* (antimicrobial susceptibility results are presented in Table 1). Next-generation sequencing (NGS) of blood and cerebrospinal fluid identified *K. pneumoniae* sequences and detected virulence markers including Aerobactin, RmpA, and the Sal fragment, confirming infection with a hypermucoviscous, hypervirulent strain (hvKp) (Table 2).

**Table 1:** Antibiotic susceptibility profile of the *Klebsiella pneumoniae* isolate.

Antibiotic	MIC (µg/ml)/ KB (mm)	Sensitivity	Antibiotic	MIC (µg/ml)/ KB (mm)	Sensitivity
Cloxacillin /Sulbactam	20*	S	Ceftriaxone	26*	S
Cefazolin	20*	I	Cefuroxime (Injection)	20*	S
Ceftazidime /Avibactam	26*	S	Amikacin	<2	S
Aztreonam	<1	S	Cefoperazone /Sulbactam	<8	S
Ciprofloxacin	<0.25	S	Cefepime	<0.12	S
Imipenem	<0.25	S	Levofloxacin	<0.12	S
Meropenem	<0.25	S	Minocycline	<1	S
Trimethoprim /Sulfamethoxazole	<20	S	Ceftazidime	<0.12	S
Ticarcillin /Clavulanate	<8	S	Tobramycin	<1	S
Piperacillin /Tazobactam	<4	S	Tigecycline	<0.5	S
Colistin	1	S	Doxycycline	1	S

I = Intermediate, K-B = Kirby-Bauer, MIC = Minimum Inhibitory Concentration, S = Sensitive;

* : Antimicrobial susceptibility testing was performed using the Kirby-Bauer disk diffusion method.

**Table 2:** Next-generation sequencing (NGS) results.

Sample	Genus			Species		Virulence gene
	Name	Sequence count	Relative abundance	Name	Sequence count	
blood	Klebsiella	14,690	89.32%	Klebsiella pneumoniae	14,506	Aerobactin、RmpA、Sal
Cerebrospinal fluid	Klebsiella	3543	96.96	Klebsiella pneumoniae	1294	not detected

The patient was preliminarily diagnosed with KPIS (with liver, lung, and brain abscesses), sepsis, and diabetic ketoacidosis.

### 2.4. Therapeutic intervention

The patient was started on meropenem (2 g every 8 hours via extended infusion over 2 hours), along with fluid resuscitation, volume expansion, glycemic control, and ofloxacin eye drops (every 8 hours). Glycemic management included continuous intravenous insulin infusion initiated at 6 to 10 U/h. Once blood glucose decreased to 11.1 mmol/L, the insulin dose was reduced to 3 to 6 U/h with concurrent intravenous glucose and normal saline. After urinary ketones cleared, insulin was transitioned to subcutaneous administration.

Within 24 hours of admission, the patient’s acid-base balance and renal function parameters normalized, with fasting and postprandial blood glucose levels stabilizing at approximately 7 mmol/L and 10 mmol/L, respectively. After 5 days of antimicrobial treatment, the patient became afebrile (37.3°C) and was successfully extubated. Repeat laboratory tests showed: WBC 9.62 × 10⁹/L, platelets 59 × 10⁹/L, neutrophils 88.30%, and procalcitonin 18.8 ng/mL. Blood cultures were negative for *K. pneumoniae*. Marked improvements in WBC, CRP, PCT, and platelet count were observed (Table 3). Follow-up CT revealed significant reduction in abscess size. Meropenem was continued for a total of 10 days, by which time all laboratory parameters had normalized.

**Table 3:** Blood parameters for the present patient.

	Day 1	Day 2	Day 3	Day 4	Day 5	Day 7	Day 14
T(°C)	38.9	39	38.8	38	37.3	37.1	36.4
WBC count (*10 ^9^L)	18.96	12.66	12.58	10.53	9.62	9.32	8.79
PCT(ng/ml)	60.35	45.88	37.19	22.73	18.8	8.37	2.11
PLT count (*10 ^9^L)	5	7	27	43	59	80	202

PCT = procalcitonin, PLT = platelet, T = highest temperature recorded each day, WBC = white blood cell.

On the second day of hospitalization, color Doppler ultrasonography revealed that the patient’s liver abscess had liquefied and was suitable for puncture. Ultrasound-guided percutaneous drainage of the liver abscess was performed, and the purulent drainage fluid was sent for laboratory analysis.

## 3. Follow-up and outcome

The patient was discharged from the ICU after 15 days, with residual right-sided weakness (muscle strength 4/5) and normal left-sided strength (5/5). At the 3-month follow-up, chest CT, abdominal ultrasound, and brain MRI showed no evidence of recurrence, indicating a favorable recovery.

## 4. Discussion

Cases of KPIS are increasingly reported across Asia, especially in Southeast Asia, with growing global recognition. In regions such as Hong Kong, Singapore, South Korea, Taiwan, and mainland China, *K. pneumoniae* has become the leading cause of pyogenic liver abscess.^[2]^ Although gas-forming liver abscesses due to *K. pneumoniae* are relatively rare (accounting for 4–24% of cases), they are associated with high mortality rates ranging from 27.7% to 37.1%.^[3,4]^*K. pneumoniae* (Kp) is a common cause of community-acquired infection. Capsular polysaccharide production is a key virulence factor that promotes bacterial survival in the host by conferring resistance to phagocytosis, complement, antimicrobial peptides, and antibodies.^[5–7]^ Capsular hyperproduction is regulated by chromosomal genes including *magA* (renamed *wzy*-K1, encoding the K1 polymerase in the *cps* locus), *rmpA*, and *rmpA2*, which regulate the hypermucoid phenotype. Notably, *rmpA* is a specific marker for hvKp due to its role in enhancing capsule production and virulence.^[8]^ The K1 serotype and *rmpA* are linked to hypermucoviscosity,^[9]^ which can be identified via the string test, a simple method with 90% accuracy for hvKp identification, though only 50% accuracy in pulmonary infections.^[10,11]^ Although serotyping is complex, a positive string test is highly suggestive of hvKp.The emergence of metagenomic next-generation sequencing (mNGS) has revolutionized pathogen detection. This unbiased, highly sensitive technique allows broad-spectrum detection of bacteria, viruses, fungi, and parasites, including rare or novel pathogens, and provides genomic data for strain tracking and resistance prediction,^[12]^ offering valuable support in clinical decision-making.

Poorly controlled type 2 diabetes is an independent risk factor for invasive KPIS in patients with community-acquired bacteremia. Hyperglycemia may promote gut colonization and endogenous liver abscess formation. Chronic hyperglycemia also induces endothelial injury, facilitating hematogenous spread and metastatic infection.^[13]^ Additionally, high glucose levels stimulate capsular biosynthesis and *cps* gene expression, increasing resistance to immune clearance.^[14]^ In this case, the patient presented with a decreased peripheral blood CD4 + T lymphocyte count, indicating an immunocompromised state. However, apart from diabetes, the patient had no history of long-term immunosuppressant use, high-dose corticosteroids, primary immunodeficiency, HIV/AIDS, or other factors known to cause immunosuppression. Thus, strict glycemic control is essential to reduce complications.Thus, strict glycemic control is essential to reduce complications.

Thrombocytopenia is a common complication in sepsis, with severity correlating with mortality. Proposed mechanisms include direct bacterial-platelet interaction, endotoxin-mediated activation, and circulating immune complexes.^[15]^ In sepsis-induced thrombocytopenia, platelets usually recover with infection control, and transfusion is generally unnecessary, though close monitoring is advised.

Historically, hvKp has been intrinsically resistant to ampicillin but susceptible to β-lactams, with a generally favorable prognosis. However, antibiotic-resistant hvKp strains are increasingly reported.^[16]^ In this case, meropenem was selected due to critical illness with sepsis, bacteremia, and brain abscess, coupled with confirmed susceptibility; low neurotoxicity and good penetration into blood, liver, brain, and lung tissues; low protein binding, making it suitable in hypoalbuminemia. Extended infusion was used to optimize time above the MIC.^[17,18]^

## 5. Study limitations

This study has several limitations. First, as a single case report, our findings are descriptive and cannot establish causality or be generalized to a broader population. The insights gained are specific to this patient’s clinical context. Second, while next-generation sequencing provided valuable virulence gene data, more extensive genomic analysis (such as whole-genome sequencing) was not performed, which could have offered deeper insights into the strain’s phylogenetic background and complete resistome. Finally, the follow-up period of 3 months, while showing no recurrence, may not be sufficient to capture potential long-term neurological sequelae or late recurrences.

## 6. Conclusion

Reports of *Klebsiella pneumoniae* invasive syndrome are increasing beyond Asia, indicating global spread. Strict glycemic control is essential to prevent disseminated complications. Antimicrobial therapy should be guided by susceptibility testing and clinical response. Next-generation sequencing offers rapid and accurate pathogen identification and characterization. Abscess drainage remains a critical intervention, with percutaneous catheter drainage being the preferred approach. Increased clinical awareness and early diagnosis are crucial for improving outcomes in this insidious and potentially severe syndrome.

## Author contributions

**Data curation:** MingZhi Fan.

**Investigation:** BoLin Wang, Fei-Hu Zhang, Hao Zhao, Peng-Cheng Li.

**Writing – original draft:** BoLin Wang.

**Writing – review & editing:** Lei Zhou.
